# Critical Role of PI3K/Akt/GSK3β in Motoneuron Specification from Human Neural Stem Cells in Response to FGF2 and EGF

**DOI:** 10.1371/journal.pone.0023414

**Published:** 2011-08-24

**Authors:** Luis Ojeda, Junling Gao, Kristopher G. Hooten, Enyin Wang, Jason R. Thonhoff, Tiffany J. Dunn, Tianyan Gao, Ping Wu

**Affiliations:** 1 Department of Neuroscience and Cell Biology, University Of Texas Medical Branch at Galveston, Galveston, Texas, United States of America; 2 Department of Neurosurgery, University of Florida, Gainesville, Florida, United States of America; 3 West China School of Preclinical and Forensic Medicine, Sichuan University, Chengdu, Sichuan, China; 4 Markey Cancer Center and Department of Molecular and Cellular Biochemistry, University of Kentucky, Lexington, Kentucky, United States of America; The University of Hong Kong, China

## Abstract

Fibroblast growth factor (FGF) and epidermal growth factor (EGF) are critical for the development of the nervous system. We previously discovered that FGF2 and EGF had opposite effects on motor neuron differentiation from human fetal neural stem cells (hNSCs), but the underlying mechanisms remain unclear. Here, we show that FGF2 and EGF differentially affect the temporal patterns of Akt and glycogen synthase kinase 3 beta (GSK3β) activation. High levels of phosphatidylinositol 3-kinase (PI3K)/Akt activation accompanied with GSK3β inactivation result in reduction of the motor neuron transcription factor HB9. Inhibition of PI3K/Akt by chemical inhibitors or RNA interference or overexpression of a constitutively active form of GSK3β enhances HB9 expression. Consequently, PI3K inhibition increases hNSCs differentiation into HB9^+^/microtubule-associated protein 2 (MAP2)^+^ motor neurons in vitro. More importantly, blocking PI3K not only enhances motor neuron differentiation from hNSCs grafted into the ventral horn of adult rat spinal cords, but also permits ectopic generation of motor neurons in the dorsal horn by overriding environmental influences. Our data suggest that FGF2 and EGF affect the motor neuron fate decision in hNSCs differently through a fine tuning of the PI3K/AKT/GSK3β pathway, and that manipulation of this pathway can enhance motor neuron generation.

## Introduction

Neural stem cells (NSCs) can self-renew and differentiate into all three neural lineages (neurons, astrocytes and oligodendrocytes). The final fate that NSCs will adopt depends on the activation of specific signaling pathways, which are stimulated by different ligands, including growth factors [1]–[3]. Among those growth factors, basic fibroblast growth factor (bFGF or FGF2) plays important roles in the development of the central nervous system, neuronal repair and survival, proliferation and neurogenesis in the developing cerebral cortex, and other neural related functions [4]–[8]. In addition, FGF2 has been extensively used for the growth of rodent, primate and human NSCs (hNSCs) in culture. Another growth factor commonly used in culturing hNSCs is epidermal growth factor (EGF). Besides their proliferation role, the two growth factors also modulate the plasticity of hNSCs [9]. Human NSCs do not generate spinal motor neurons (MNs) after differentiation. However, when hNSCs are primed in the presence of FGF2, they express a higher level of MN-specific transcription factor HB9 and generate more MNs than after priming with EGF, leukemia inhibitory factor (LIF) or EGF plus LIF. Thus, exposure to distinct growth factors elicits different cell fates. Interestingly, FGF2 and EGF activate some common signaling pathways [10], which raises the question on how these factors lead to different phenotypic outcomes. One possibility is that these different growth factors activate the same canonical signaling but different non-canonical pathways [11]. A second possibility is that they activate the same pathways but with different intensities of activation (e.g. phosphorylation level of downstream proteins) or different duration of activation (e.g. the time that the proteins in the pathway remain phosphorylated) [12]. Knowledge of how these factors control MN differentiation from NSCs may have profound significance in therapies for MN disorders, such as amyotrophic lateral sclerosis and spinal cord injury, where MN replacement may be one of the ultimate choices. Unfortunately in many studies using stem cell transplantation, the only benefits observed are due to stem cell-released trophic factors or decreased inflammation that promotes survival of endogenous MNs [13]–[16]. On the other hand, several groups including us have obtained spinal MNs from either embryonic stem cells or neural stem cells of human or rodent origin [17]–[19]. However, the percentages of stem cells becoming MNs vary from 0.5% to 50% [17], and the underlying molecular mechanisms controlling MN fate specification in response to different growth factors remain elusive.

In this study we asked how FGF and EGF differentially influence the same cell signaling pathways on the same target cells (hNSCs) to achieve different fates. Surprisingly, we found that the PI3K (phosphatidylinositol 3-kinase)/Akt/GSK3β (Glycogen synthase kinase 3β) pathway modulates the decision of hNSCs to become MNs, both in vitro and after transplantation into the rat spinal cord, even in areas that normally do not possess MNs. This discovery could provide insights into stem cell-based therapies to replace MNs lost in diseases.

## Results

### FGF2 and EGF differentially affect the temporal pattern of Akt and GSK3β phosphorylation in hNSCs

**Figure 1:**
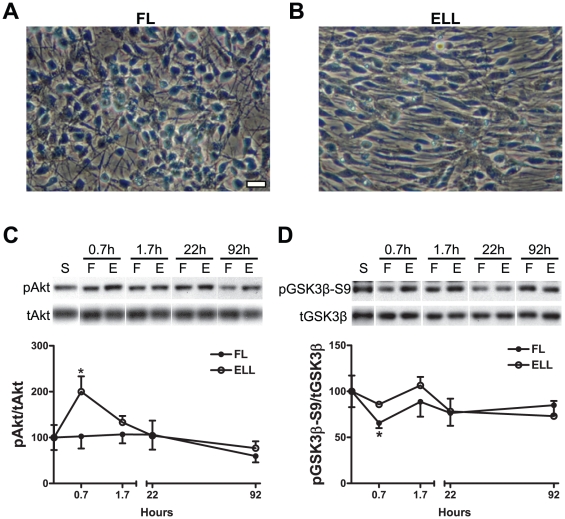
FGF or EGF priming exhibits different temporal patterns of signal activation in hNSCs. (A–B) Representative phase contrast microscope images show hNSCs primed in either FL (A) or ELL (B) for 4 days. Scale bar = 25 µm. (C–D) Temporal patterns of signal phosphorylations in FL-primed vs. ELL -primed hNSCs at different time points assessed by Western blot analyses. Densitometric values, depicted as a ratio of the phosphorylated form over total amount of the specific protein, are plotted for phospho-Akt Ser473 (C) and phospho-GSK3β (Ser 9) (D). Data are presented as mean ± SEM (n = 3); * p<0.05, two-way ANOVA.

We have previously discovered that priming with FGF2 plus laminin (FL) endowed hNSCs with the potential to differentiate into MNs, whereas EGF/laminin or EGF plus LIF/laminin (ELL) priming inhibited MN formation [9]. LIF alone showed no effect. The phenotypic differences between FGF2 and EGF were accompanied by different levels of Akt phosphorylation at the end of the 4-day priming period [9]. It is known that activation of the FGF and EGF receptors, FGFR and EGFR, respectively, could both lead to the activation of the PI3K/Akt pathway that negatively regulates GSK3β [20]. However, it is unknown whether the different levels of Akt activation contribute to the differential MN generation of hNSCs in response to FGF2 and EGF. Thus, we first compared the temporal phosphorylation pattern of the Akt signaling pathway in hNSCs during FL or ELL priming. FL- and ELL-primed cells, in addition to the morphological differences (uniform and multipolar in FL vs. heterogeneous and spindle shape in ELL, Fig. 1A–B), showed different phosphorylation patterns of Akt and GSK3β (Fig. 1C–D). The 40-min (0.7 hr) was chosen as the earliest time point in this study to ensure the attachment of hNSC neurospheres required for priming. While FL priming consistently maintained lower levels of phosphorylated Akt Ser473, ELL promoted higher phosphorylation of Akt during the first two hours of priming (Fig. 1C) and thus increased Akt's activity. The latter was further evidenced by a higher phosphorylation of the downstream GSK3β Ser9 (Fig. 1D). In contrast, FL reduced GSK3β-Ser9 phosphorylation that would increase its activity, especially during the early phase of priming. We also analyzed the phosphorylation of GSK3β at Tyr216 (which activates GSK3β activity), and found it being phosphorylated in both priming treatments (data not shown). Thus, GSK3β is negatively regulated by Akt via Ser9 phosphorylation, with ELL priming evoking less GSK3β activity than FL priming.

### High levels of PI3K/Akt activation inhibit MN differentiation from hNSCs

Since the temporal activation patterns of the examined kinases were different between FL and ELL (Fig. 1), we asked if their activities have any impact on the MN induction from hNSCs. To this end, we systematically inhibited these kinases and analyzed the effects of the inhibitions on the expression of the MN transcription factor HB9. The initial focus was to disrupt the PI3K/Akt pathway using both RNA interference (RNAi) of PI3K and chemical inhibitors against PI3K and Akt.

**Figure 2:**
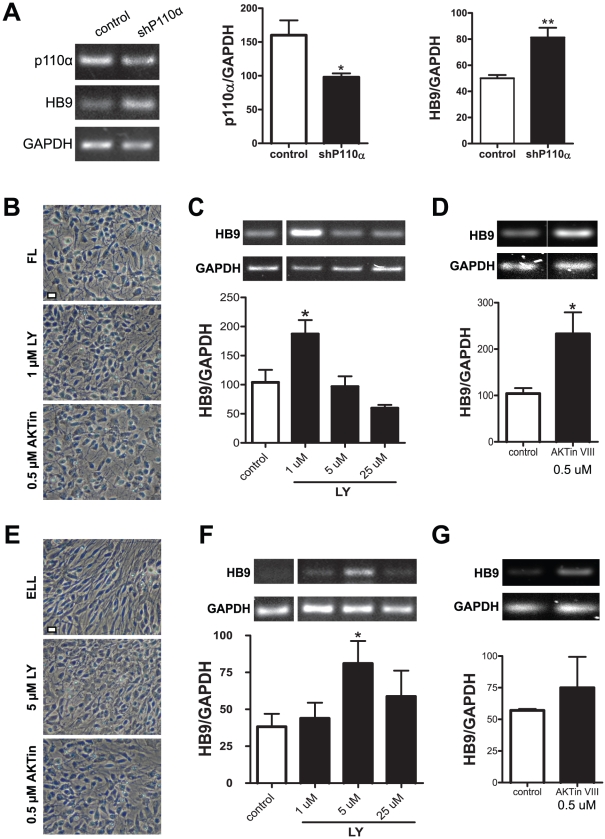
Blocking PI3K/Akt signaling by RNAi or inhibitors enhances HB9 mRNA expression in hNSCs. (A) Effect of PI3K knockdown on HB9 expression in hNSCs. Cells were transfected with either an empty pLK0.1 vector (control) or with pLK0.1 containing DNA coding for a shRNA targeting the p110α catalytic subunit of PI3K (shP110α). Semi-quantitative RT-PCRs showed that shP110α decreased p110α and increased HB9 transcripts. (B–D) Effect of PI3K/Akt inhibition on hNSCs primed for 4 days in FL plus DMSO (FL or Control). Cells were treated with inhibitors for PI3K (LY294002, 1–25 µM) or Akt (Akt inhibitor VIII, 0.5 µM). Treatment with LY294002 at 1 µM (B–C) or Akt inhibitor VIII at 0.5 µM (B and D) did not change the cell morphology (B), but increased HB9 transcripts compared with the control group (C–D). (E–G) Effect of PI3K/Akt inhibition on hNSCs primed for 4 days in ELL plus DMSO (ELL or Control). (E) Representative images show that, in the presence of these inhibitors, hNSCs lost the spindle and/or radiated cell shape seen in the ELL control. Treatment with LY294002 (5 µM) (F) and Akt inhibitor VIII (0.5 µM) (G) increased HB9 transcript levels compared with the control group. Densitometric values were normalized to GAPDH internal controls and are presented as mean ± SEM (n = 3); * p<0.05, ** p<0.01; one-way ANOVA or *t* test. Scale bars = 25 µm.

Human NSCs were transfected with vectors containing short hairpins (shRNAs) that target either the p110α or p110β catalytic subunits of PI3K. Three days later, hNSCs were primed for four days with FL, followed by three days in B27 differentiation media. Semi-quantitative RT-PCRs showed that p110α shRNA decreased the expression p110α by 39%, which correlated with a 77% increase in HB9 mRNA (Fig. 2A). Interestingly, a significant knockdown of p110β RNA by shRNA had no effect on HB9 levels (Fig. S1A). Thus, PI3K activity, particularly the p110α subunit, negatively influenced the expression of HB9 in hNSCs. To verify these RNAi results, hNSCs were treated with a specific PI3K inhibitor, LY294002 at various concentrations (1 to 25 µM) based on its IC_50_ (1.4 µM). Addition of the inhibitor at 1 µM during all 4 days of FL priming did not change the overall morphology of hNSCs (Fig. 2B), but resulted in an 80% increase in HB9 mRNA expression (Fig. 2C). Similar results were also seen with 0.2 µM LY294002 (data not shown). However, higher doses of LY294002 (5 and 25 µM) resulted in decreased HB9 expression, which could be due to toxic effects of excess LY294002 as indicated by the WST-1 cell viability assay (Fig. S1B). Cells treated with another PI3K inhibitor, wortmannin, also displayed an increase in HB9 mRNA (Fig. S1C), which was accompanied by a decrease in the phosphorylation of Akt at Ser473 (a PI3K downstream target) (Fig. S1C) and a reduction of phosphorylated GSK3β-Ser9. Together, these data demonstrate that PI3K activation reduces HB9 expression in hNSCs.

We then investigated the effect of inhibiting the PI3K downstream target, Akt, during FL priming. Akt inhibitor VIII at 0.5 µM significantly increased the expression of HB9 by 1.2-fold (Fig. 2D). Akt inhibitor V also caused a dose-dependent enhancement of HB9 expression, albeit statistically insignificant (Fig. S1E). These data suggest that the observed effect of PI3K inhibition on HB9 was probably due to the subsequent Akt inhibition. To confirm that the higher PI3K and Akt activation levels in ELL were responsible for the decreased HB9 transcripts, we applied the same inhibitors used for FL during ELL priming. Noticeably, the base-line expression of HB9 in ELL-primed cells (around 35–55 units, Fig. 2F–G) was much lower than that in FL-primed (above 100 units, Fig. 2C–D), confirming the inhibitory role of EGF in motor neuron differentiation as we reported previously [9]. Higher concentrations (5 µM) of LY294002 was required to increase HB9 expression by 1.1-fold (Fig. 2F), along with significant changes of cell morphology and migration pattern that resembled what was observed in FL-primed cells (Fig. 2E). Furthermore, Akt inhibitor VIII also caused an increase of HB9 in ELL cells (Fig. 2G). The differential doses of LY294002 needed to enhance HB9 gene expression under different priming conditions correlated well to their effects on blocking Akt activities. As shown in Fig. S1F–G, the basal level of phosphorylated Akt (pAkt) in FL was lower than that of ELL-primed cells. One µM of LY294002 sufficiently reduced further the Akt phosphorylation in FL cells, whereas 5 µM was required to produce a similar effect in ELL-primed cells. There were no changes in the levels of total Akt among different treatments (Fig. S1H).

### PI3K/Akt-mediated decrease of HB9 expression correlates with an increase of GSK3β Ser9 phosphorylation

**Figure 3:**
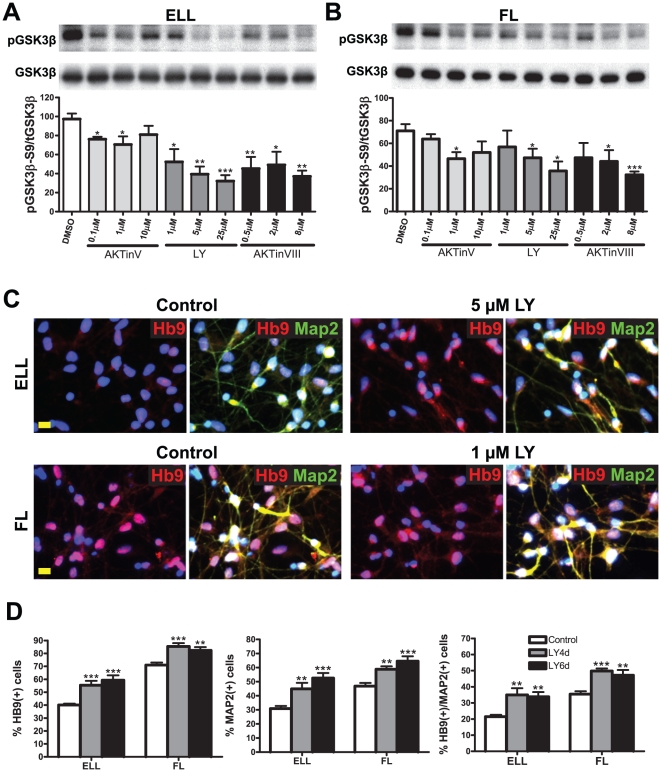
Inhibition of PI3K/Akt activity reduces phosphorylation of GSK3β while increases HB9 motor neuron differentiation. (A–B) Effect of PI3K/Akt inhibition on GSK3β phosphorylation in hNSCs primed for 40 minutes in either ELL (A) or FL (B). Cells were treated during priming with the PI3K inhibitor LY294002 or the Akt inhibitors V or VIII, and analyzed by western blot densitometry for the phosphorylation status of GSK3β at Ser9. Phospho GSK3β at Ser9 (pGSK3β-S9) values were normalized to total GSK3β (tGSK3β). In both ELL and FL primed groups, all inhibitors decreased GSK3β Ser9 phosphorylation as compared with DMSO controls (mean ± SEM; n = 3; * p<0.05, ** p<0.01, *** p<0.001, one-way ANOVA). (C–D) Effect of PI3K inhibition on motor neuron differentiation. Cells were treated with either DMSO (control) or 1–5 µM LY294002 for four days during priming (C and LY4d in D) or 4-day priming plus 2-day B27 differentiation (LY6d in D). Representative images are shown in (C) for immunostaining using antibodies against HB9 (red) and microtubule-associated protein 2 (MAP2; green). Nuclei were counterstained with DAPI (blue). Scale bars = 50 µm. Quantitative analyses show that LY294002 treatments increased the percentages of HB9^+^, MAP2^+^ and double-labeled (HB9^+^ MAP2^+^) motor neurons in both ELL and FL groups (D). Data are the mean ± SEM, n = 3. ** p<0.01 and *** p<0.001, one-way ANOVA.

To further understand how PI3K/Akt inhibition increased mRNA levels of HB9, we exposed cells primed in either ELL (Fig. 3A) or FL (Fig. 3B) to the PI3K inhibitor LY294002 or the Akt inhibitors V or VIII, and analyzed the phosphorylation status of GSK3β Ser9, a major downstream target of Akt. After 40 minutes of priming, proteins were extracted for Western blot analyses. As expected, all the inhibitors decreased the phosphorylation of GSK3β Ser9 (Fig. 3A–B), thus increasing GSK3β activity. We also analyzed the MN differentiation pattern of these cells. Human NSCs were subjected to either ELL or FL priming for four days, followed by a ten-day differentiation period in B27 media. In the control groups, there were very few ELL-primed cells with strong HB9 immunoreactive labeling, while most of FL cells (over 70%) were strongly labeled with HB9 (Fig. 3C). Treating ELL- or FL-primed cells with LY294002 resulted in higher numbers of HB9^+^ cells and of MNs (as assessed by the coexpression of a pan-neuronal marker, microtubule-associated protein 2 or MAP2, and the MN specific marker HB9, Fig. 3C). Cell counts (Fig. 3D) confirmed that the numbers of total neurons (MAP2^+^) and of specific MNs (HB9^+^/MAP2^+^) increased after LY294002 treatment for 4 days in both ELL and FL groups. Prolonged treatment with LY294002 for 6 days increased the percentage of MAP2^+^ cells in both groups without changes in HB9^+^/MAP2^+^ MNs (Fig. 3D).

### GSK3β activation is required for MN differentiation from hNSCs

**Figure 4:**
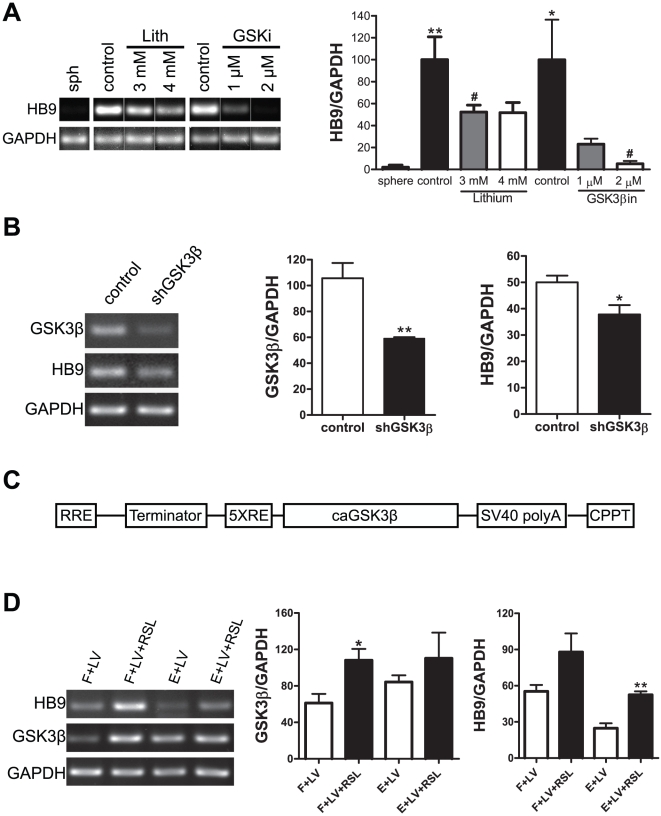
GSK3β activation is required for HB9 mRNA expression in hNSCs. (A) Effect of GSK3β inhibition on HB9 expression in hNCSc. Cells were exposed to the GSK3β inhibitors (lithium and GSK3β-inhibitor VIII) during FL priming for 4 days. Semi-quantitative RT-PCRs showed that FL priming (control) increased HB9 expression as compared to spheres (* p<0.05, ** p<0.01), while both inhibitors decreased HB9 transcript levels as compared to vehicle controls (# p<0.05). (B) Effect of GSK3β knockdown on HB9 expression in hNSCs. Cells were transduced with a lentiviral vector containing a short hairpin that targets GSK3β (shGSK3β), primed with FL for 4 days and then differentiated for 3 days in B27. Semiquantitative RT-PCRs showed that shGSK3β decreased both GSK3β and HB9 transcripts as compared with the vector control. (C) Schematic illustration of an engineered inducible lentiviral vector (LVcaGSK3β), which contains RRE (Rev response element), Terminator (SV40 terminator), 5XRE (GAL4 response element), caGSK3β (constitutive active mutant form of the GSK3β, Ser9Ala), SV40 polyA signal, CPPT (central polypurine tract). (D) Effect of induced overexpression of constitutively active GSK3β on HB9 expression in hNSCs. An hNSC cell line expressing the RheoReceptor-1 and RheoActivator was transduced with LVcaGSK3β (LV) and primed in either FL (F+LV) or ELL (E+LV). Treatment with synthetic ligand RSL-1 increases HB9 mRNA. GSK3β or HB9 densitometric values were normalized to GAPDH internal controls, and are presented as mean ± SEM; n = 3; * p<0.05, ** p<0.01, # p<0.05; one-way ANOVA or *t* test.

Since inhibiting the PI3K/Akt cascade resulted in decreased phosphorylation of GSK3β Ser9 and increased MN differentiation from hNSCs, we then asked if the increase in HB9 expression was due to the increase in GSK3β activity. To this end, we exposed hNSCs to the GSK3β inhibitors, lithium and GSK3β-inhibitor VIII, during FL priming; and found that both inhibitors decreased HB9 (Fig. 4A). Lithium at 3–4 mM and 2 µM GSK3β-inhibitor VIII reduced HB9 by 50% and 94%, respectively, without causing cytotoxicity (Fig. S1B). Furthermore, we used a lentiviral vector containing a short hairpin for GSK3β (shGSK3β). Two to three days after exposure to shGSK3β at the multiplicity of infection (MOI) of 1, hNSCs were primed with FL for four days and then differentiated for three days in B27, the time point exhibiting the peak HB9 mRNA expression based on our previous report [9]. Semiquantitative RT-PCRs showed that, comparing to the vector control, shGSK3β significantly decreased the expression of GSK3β by 44% and reduced the expression of HB9 by 24% (Fig. 4B).

To determine whether increased GSK3β activity resulted in an enhancement of HB9 expression, we engineered a lentiviral vector (LVcaGSK3β) (Fig. 4C) containing a constitutive active mutant form of the GSK3β (caGSK3β) under the control of an inducible promoter [GAL4 response element (5XRE)], which is regulated by the synthetic RSL1 ligand when two other proteins (the RheoReceptor-1 and activator) are also present. A stable cell line of hNSCs was first created by transfecting cells with the RheoSwitch plasmid pNEBR-R1 containing RheoReceptor-1 and RheoActivator. The efficiency of this RheoSwitch inducible system was tested in hNSCs by transfecting these cells with a plasmid containing the Gaussia princeps secreted luciferase (GLuc) reporter under the control of the 5XRE promoter. As shown in Fig. S2, the hNSC cell line secreted negligible luciferase in the absence of RSL-1, while adding RSL-1 increased the secretion by more than 100-fold, indicating the RheoSwitch inducible system was highly efficient with minimal leakage. The same cell line was then transduced with LVcaGSK3β at the MOI of 1. RSL1 ligand lead to a significant increase of GSK3β transcription in FL, and to an enhancement of HB9 expression in both FL (60%, p = 0.059) and ELL (1.2 fold) groups (Fig. 4D). Consequently all these data suggests that the fact that FL priming increases HB9 levels whereas ELL priming decreases its expression could be explained, at least in part, by their differential effects on GSK3β activity.

### Manipulation of PI3K/Akt/GSK3β affects the expression of other MN lineage-related transcription factors

**Figure 5:**
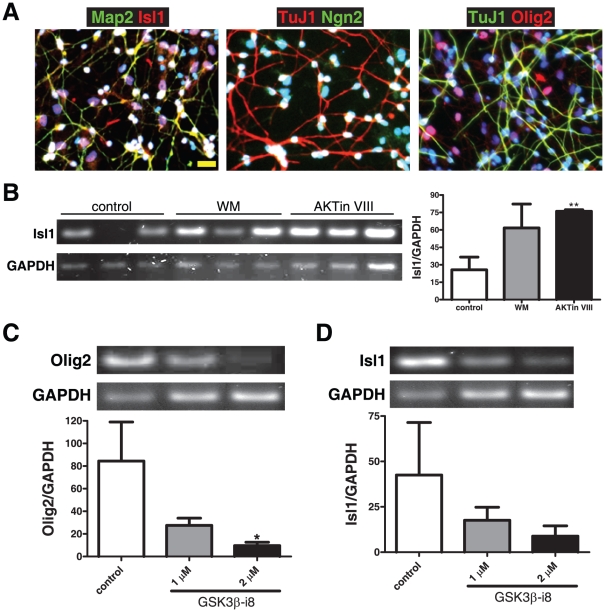
Effect of PI3K/Akt/GSK3β inhibition during FL priming on other motor-neuron linage-related transcription factors. (A) Representative images of FL-primed and differentiated hNSCs detected by immunofluorescent staining with antibodies against microtubule-associated protein 2ab (MAP2), Isl1, class III β-tubulin (TuJ1), Ngn2, Olig2. Nuclei are counterstained with DAPI. (B) Effect of PI3K/Akt inhibition on Isl1 mRNA expression in hNSCs. Cells were primed for four days in FL and treated with inhibitors for PI3K (wortmannin, 1 µM) or Akt (Akt inhibitor VIII, 0.5 µM). Both treatments increased Isl1 transcripts compared with the control group. Densitometric values were normalized to GAPDH internal controls and are presented as mean ± SEM (n = 3); ** p<0.01, one-way ANOVA. (C–D) Effect of GSK3β inhibition on Olig2/Isl1 mRNA expression in hNSCs. Cells were exposed to two doses (1 and 2 µM) of GSK3β-inhibitor VIII (GSK3β-i8) during FL priming for four days. Semi-quantitative RT-PCR showed that this inhibitor decreased Olig2 (C) and Isl1 (D) transcript levels as compared to controls. Data are mean ± SEM, n = 3, * p<0.05, one-way ANOVA.

Besides HB9, we also analyzed the effect of inhibiting the PI3K/Akt/GSK3β pathway on the expression of other MN-related transcription factors, such as Olig2, Isl1 and Ngn2. Olig2 is an early transcription factor for the MN-oligodendrocyte cell lineage. It is required for the expression of other early MN transcription factors such as Ngn2 and Lhx3, but antagonizes the premature expression of later transcription factors such as HB9. Isl1, although also expressed in interneurons, is a LIM homeodomain transcription factor that directly sits on the HB9 promoter and activates its transcription. FL priming is also capable of fostering the expression of these transcription factors. Immunofluorescent staining revealed that many of the hNSCs, after FL-priming and differentiation in B27 for nine days, became MAP2^+^ Isl1^+^ or Tuj1^+^Ngn2^+^ neurons (Fig. 5A).

Semiquantitative RT-PCR analyses showed that Olig2 levels were not affected in the wortmannin- and Akt inhibitor VIII-treated groups (data not shown). Isl1 expression, on the other hand, displayed a similar pattern as HB9, i.e., increasing in levels when cells were exposed to either wortmannin or Akt inhibitor VIII (Fig. 5B). In addition, we found that inhibition of GSK3β with GSK3β-inhibitor VIII decreased both Olig2 and Isl1 (Fig. 5C–D). No changes were detected in the expression levels of Otx2 (a forebrain and midbrain transcription factor) and Pax6 (a transcription factor for the ventral spinal cord) in primed hNSCs after treatment with these PI3K/Akt inhibitors (data not shown).

### PKC-ζ activation is necessary for MN differentiation from hNSCs

**Figure 6:**
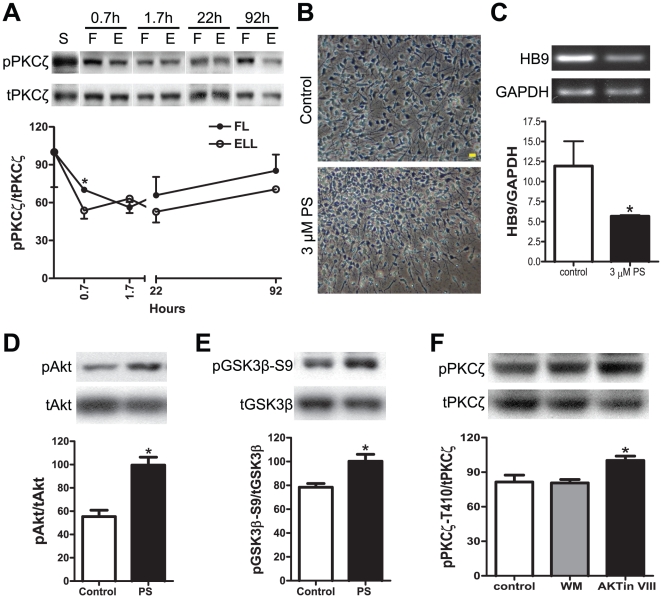
PKC-ζ activation is required for HB9 mRNA expression in hNSCs. (A) Temporal patterns of PKC-ζ phosphorylation in FL-primed vs. ELL-primed hNSCs at different time points assessed by Western blot analyses. (B) Representative phase contrast images of FL-primed hNSCs treated with 3 µM PKC-ζ pseudosubstrate inhibitor (PS) for four days. Note the complete spread of cells into monolayer in the FL-priming group (Control), in contrast to the limited spread of cells away from spheres in the PS-treated group (PS). Scale bar = 20 µm. (C) Effect of PKC-ζ inhibition on HB9 expression in hNSCs. Semi-quantitative RT-PCR and densitometric analysis show that PS decreases HB9 expression as compared with the control. HB9 values were normalized to GAPDH internal controls. (D–E) Effect of PKC-ζ inhibition on Akt/GSK3β activity in hNSCs. Cells were FL-primed and treated with either water (control) or the PKC-ζ pseudosubstrate inhibitor (PS) for 3 days. Densitometric values are shown for pAkt (D) and pGSK3β-S9 (E). (F) Effect of PI3K/Akt inhibition on PKC-ζ phosphorylation in hNSCs. Cells were FL-primed and treated with either DMSO (control), 1 µM wortmannin (WM) or 0.5 µM Akt inhibitor VIII (AKTin VIII) for 3 days. Data are presented as mean ± SEM (n = 3); * p<0.05, one-way ANOVA or *t* test.

The above studies indicated that activation of PI3K, via Akt, negatively regulates MN differentiation from hNSCs. Since PI3K also activates other targets, including PKC-ζ (protein kinase C zeta, an atypical PKC) by phosphorylating it at Thr410 [21], we asked whether the status of PKC-ζ activation affected MN fate determination. Unlike Akt (Fig. 1C), phosphorylation of PKC-ζ at Thr410 exhibited an increased temporal pattern of activation in the FL group as compared with the ELL group (Fig. 6A). To determine whether PKC-ζ activity was required for the FL induction of HB9, we exposed hNSCs to the PKC-ζ specific inhibitor (myristoylated pseudosubstrate inhibitor or PS, Table S1) during FL priming. Three µM PS dramatically decreased the migratory capacity of hNSCs, showing the cells stayed near the center of the spheres (Fig. 6B), as wells as reduced the HB9 transcripts (Fig. 6C).

Next, we tested whether there was a connection between PKC-ζ and Akt/GSK3β with respect to HB9 transcription. Interestingly, blocking the activity of PKC-ζ by PS increased phosphorylation of both Akt Ser473 and GSK3β Ser9 (Fig. 6D–E), suggesting that maintaining PKC-ζ activity is required to suppress the high-level activation of Akt and subsequently allows GSK3β to function properly in MN differentiation. Similarly, we assessed the phosphorylation status of PKC-ζ after priming hNSCs in FL for 3 days in the presence of PI3K or Akt inhibitors. Western blot analyses showed that 1 µM Wortmannin (a concentration enhanced HB9) did not change the level of PKC-ζ phosphorylation at Thr410 (Fig. 6F). In contrast, inhibition of Akt activity by Akt inhibitor VIII significantly increased phosphorylated PKC-ζ (Fig. 6F). Taken together, these results identified a negative interplay between PKC-ζ and Akt in FL-primed hNSCs, and showed that the required activity of PKC-ζ for HB9 expression was PI3K-independent.

Besides the atypical PKC-ζ, the PKC family comprises other isozymes grouped into classical and novel PKCs (cPKCs and nPKCs respectively). FGF and EGF can also activate these PKCs through phosphorylation of phospholipase C gamma (PLC-γ) [22]. Analysis of the temporal pattern of activation of these kinases showed no differences between FL and ELL when using a pan antibody against all phosphorylated PKCs (data not shown). Furthermore, a specific PLC-γ inhibitor (U73122) and an inhibitor for both classical and novel PKC (GF 109203X, Table S1), did not affect HB9 expression in FL-primed cells (data not shown). Thus, the activation of other conventional and novel PKC isoforms downstream of PLC-γ does not seem to contribute to FGF-induced HB9 expression, whereas PKC-ζ activity is required for this induction.

### Blocking PI3K/Akt activity increases MN differentiation from transplanted hNSCs in vivo

**Figure 7:**
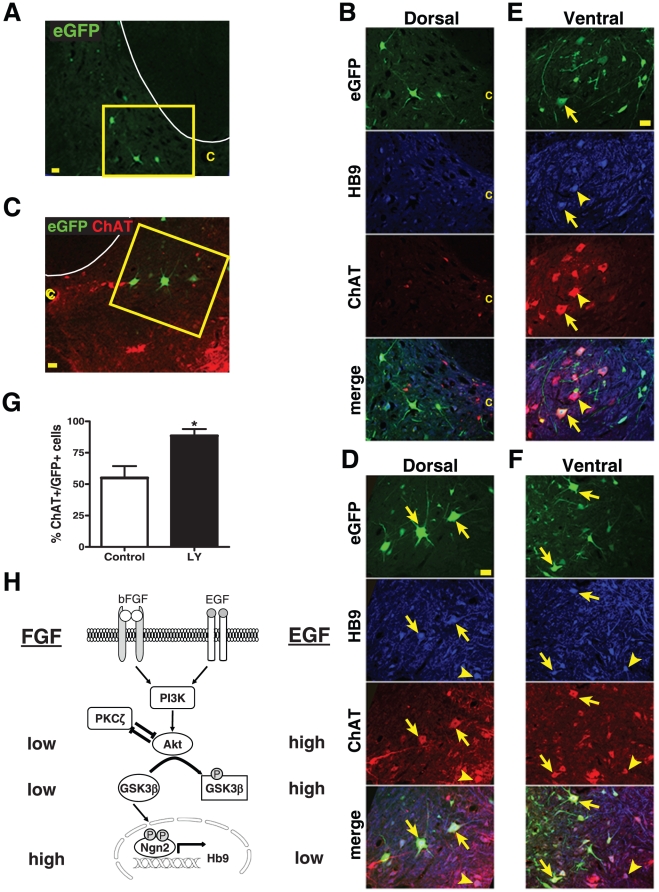
Blocking PI3K signaling increases motor neuron differentiation in hNSCs grafted into adult rat spinal cords. (A–B) Representative confocal images of enhanced green fluorescent protein (eGFP)-labeled hNSCs in the dorsal horn of the adult rat spinal cord L4 at 1 month post transplantation. White curve outlines the dorsal horn in (A). (B), higher magnification images of the inset in (A). Grafted hNSCs with FL-priming alone did not co-label with choline acetyltransferase (ChAT, red) or HB9 (blue). (C–D) FL-primed hNSCs, when treated with LY294002, differentiated into Hb9/ChAT immunoreactive motor neurons in the Lamina VII of rat spinal cord. (D), higher magnification images of the inset in (C). (E) FL-primed hNSCs differentiated into ChAT^+^/Hb9^+^ motor neurons in the ventral horn. (F) FL-primed and LY294002-treated hNSCs differentiated into Hb9^+^/ChAT^+^ motor neurons in the ventral horn. (G) Cell counts show a significant increase of the percent of ChAT^+^ neurons from the grafted eGFP^+^ hNSCs treated with LY294002 when compared to the control. Scale bars = 50 µm. C, central canal. Arrow, grafted eGFP^+^ hNSCs become Hb9^+^/ChAT^+^ motor neurons. Arrowhead, endogenous Hb9^+^/ChAT^+^ motor neurons. (H) Proposed model for differential effects of FGF and EGF on motor neuron differentiation from hNSCs. FGF and EGF, via activating their receptor tyrosine kinases (FGFR and EGFR respectively), differentially affect the PI3K/Akt/GSK3β pathway. FGFR-induced PI3K/Akt activation is lower than EGFR, which results in a lower phosphorylation of GSK3β at Ser 9 with increased activity. Higher GSK3β activity leads to an enhanced transcription of HB9, presumably via Ngn2 phosphorylation/activation. PKC-ζ cross-talks with Akt through mutual inhibition, which then modulates GSK3β activity and HB9 expression.

The above studies suggested a negative impact of PI3K/Akt activation on MN specification from human fetal NSCs *in vitro*. We then investigated whether blocking the PI3K signals could enhance MN differentiation from the hNSCs grafted into adult rat spinal cords. Human NSCs treated with or without 1 µM LY294002 (LY) during FL priming were injected into the L4 ventral horn (n = 9 per group). To track the grafted cells in rat spinal cord, hNSCs were pre-labeled with enhanced green fluorescent protein (eGFP) via recombinant adeno-associated viral (AAV) vector transduction. Morphological analyses revealed that many grafted cells acquire neuronal characteristics in both dorsal and ventral horns of the spinal cords at one month post-transplantation (Fig. 7). Some large neuron-like grafted cells (green) were found in dorsal horns from both the control group (Fig. 7A–B) and the LY-treated group (Fig. 7C–D, Fig. S3). However, only LY-treated cells became HB9^+^/choline acetyltransferase (ChAT)^+^ MNs (12.5%), some in diameter near 50 µm, in Laminae V–VI (Fig. S3) and VII (Fig. 7D), where there are either no endogenous ChAT^+^ neurons or no ChAT^+^ neurons larger than 25 µm. In contrast, FL-primed cells without LY treatment expressed neither HB9 nor ChAT (Fig. 7B) in the dorsal region. In ventral horns, about 55% of GFP-expressing cells were co-labeled with HB9 and ChAT in animals receiving FL-primed hNSCs (Fig. 7E and G), while 88.5% of FL-LY-treated hNSCs became HB9^+^/ChAT^+^ cells (Fig. 7F–G). Therefore, LY-treatment significantly increased MN differentiation from grafted hNSCs in adult rat spinal cord.

## Discussion

The present work demonstrated a novel role of the PI3K/Akt/GSK3β pathway in spinal MN specification from hNSCs in response to FGF2 or EGF. We showed for the first time that manipulation of this pathway significantly influenced the fate of hNSCs to become MNs, both in vitro and after transplantation into adult rat spinal cords.

Stem cells may be a source to replace MNs that are lost in diseases such as amyotrophic lateral sclerosis and spinal cord injury. Previously many groups, including ours, have successfully generated MNs from embryonic or neural stem cells [17]. We further discovered that priming hNSCs with FGF2-containing media (FL) potentiated them to become MNs, while priming with EGF-containing media (ELL) drove hNSCs away from the MN differentiation path. Instead these ELL-primed cells differentiated toward glutamate and γ-aminobutyric acid (GABA) neuronal subtypes [9]. As a first step to elucidate how molecular signaling differences triggered by these two growth factors result in a different cell fate (i.e. MN), we compared the major canonical pathways activated by FGF and EGF and found that they share similar signals, including Ras/MAPK, PI3K/Akt and PLC-γ/PKC [10]. We, therefore, focused on differences in intensity of phosphorylation of the pathway elements and the duration of this phosphorylation. Analysis of the temporal patterns of activation revealed increased activity of Akt (i.e. increased phosphorylation of Akt Ser473 and its target GSK3β Ser9) during ELL priming as compared with FL. Since Akt activation by growth factor stimulation requires PI3K activity [23], [24], we blocked PI3K using RNAi against the PI3K catalytic subunits p110α or p110β. Knock-down of p110α promoted an increase in HB9 expression, while RNAi against p110β had no effect on HB9. The latter may be due to a more prevalent role of p110β in G-protein coupled receptor than in receptor tyrosine kinase signaling as previously observed [25]. In addition, applying specific PI3K chemical inhibitors increased HB9 transcript levels and decreased phosphorylation of Akt Ser473. This evidence points towards the PI3K/Akt pathway being responsible for repressing HB9 expression. However, PI3K activity affects several other molecules besides Akt [26]. In order to verify that Akt was actually involved in the regulation of HB9, we inhibited Akt directly using several inhibitors, and found that Akt inhibition increased HB9 levels, which correlated with a decrease in GSK3β phosphorylation at Ser9, a modification that decreases GSK3β activity. Further manipulation of GSK3β showed that increased active GSK3β by expressing its constitutive active mutant form enhanced HB9 expression, whereas inhibitory manipulations of GSK3β by RNAi or inhibitors decreased HB9, confirming that GSK3β was required for proper HB9 expression.

Several other transcription factors, such as Olig2, Isl1, and Ngn2, are also critical in specification of the MN lineage during development [27]–[29]. In FL-primed hNSCs, we found that most of these markers behaved in a similar way as HB9, for example, Isl1 and Ngn2 levels increased when PI3K/Akt were inhibited. Moreover, Isl1 and Olig2 were downregulated when GSK3β activity was decreased. One exception is Olig2, i.e., blocking PI3K/Akt did not affect the level of Olig2 transcript. A possible explanation is that Oligo2, unlike Isl1 and HB9, may also be regulated by other unknown molecules, in addition to the PI3K/Akt/GSK3β pathway. This makes sense since Olig2 is a critical transcription factor promoting the development of a common progenitor lineage that eventually generates both MN and oligodendrocyte [29]. However, a continuously high level of Olig2 inhibits MN while promotes oligodendrocyte differentiation. Thus, the lack of effect on Olig2 but increasing Isl1 and Ngn2 by inhibiting PI3K/Akt would act in concert to facilitate MN generation.

All these data together support a model in which PI3K and Akt activation by the stimulated EGFR (in ELL priming) is higher than the activation caused by the stimulated FGFR (in FL priming) (Fig. 7H). Higher PI3K and Akt activities result in a decreased GSK3β activity, which leads to a decreased expression of MN markers. In other words, two different growth factors, FGF2 and EGF, via their different effects on the activation of the PI3K/Akt/GSK3β pathway, result in differential MN specification of cultured hNSCs. This model agrees with a previous in vivo mouse study, which showed that for MN differentiation to proceed, Ngn2 had to be phosphorylated on two key serine residues, S231 and S234, and that GSK3β was required for this phosphorylation [30]. Activation of the PI3K pathway either positively or negatively contributes to the differentiation of various types of cells, including immune, intestinal or muscle cells [31]–[34]. We are the first to demonstrate a negative role of PI3K/Akt/GSK3β in mediating neuronal differentiation. Noticeably hNSC neurospheres are heterogeneous in nature, i.e. containing both stem cells and progenitors at various stages. They are extremely sensitive and respond differently to external cues. All these could result in variations observed among different experiments [9], and possibly contribute to the slight changes of some of the signaling molecules characterized in this study. On the other hand, the moderate differences in the levels of Akt and GSK3β phosphorylation between FL and ELL result in significant changes of MN differentiation from the heterogeneous hNSCs, as shown both by the analyses of HB9/Map2 double-labeled neurons here and of the HB9/ChAT neurons in our previous report [9]. This is not totally surprising since the fine tuning of PI3K activation is most likely critical for eliciting its multiple roles in cell proliferation, survival or differentiation. Given apparent cell death accompanied with cell differentiation following withdrawal of growth factors, and particularly after Akt inhibition, we speculate that motor neuron differentiation occurs to some extent at the expense of cell survival. Relationships among cell proliferation, death/survival, and neuronal differentiation are certainly granted for future investigation.

The question is then whether the PI3K/Akt/GSK3β pathway is the sole player to determine MN fate specification. So far, we excluded out two other canonical pathways shared by FGF and EGF, MAPK [9] and PLC-γ here, since blocking these signals did not affect HB9 expression and/or MN differentiation. In additional to these shared pathways, EGF is known to also activate the STAT3 pathway. However, STAT3 inhibition exhibited little or no effect on HB9 expression in EGF-primed cells (unpublished observations). It is noticeable that the phosphorylation patters of Akt and GSK3β, as well as their activity inhibitions, are not exactly correlated to each other. One explanation is that even though higher phosphorylation of AKT usually translates into higher phosphorylation of GSK3β, these changes may not always be proportional as other pathways (e.g. Wnt) also affect the final phosphorylation state of GSK3β in a given cell. Along this line, whether and how Wnt and/or other unknown signaling pathway(s) are also involved in MN specification remains to be determined.

To begin exploring other signaling molecules, we found that PKC-ζ was also required for HB9 expression in hNSCs. Interestingly, both PKC-ζ and Akt were previously reported downstream effectors of PI3K [21], [35]. However, in hNSCs, blocking PKC-ζ or Akt resulted in opposite effects on HB9 mRNA expression, and the phosphorylation of PKC-ζ was PI3K-independent. One possibility is that other signaling molecule(s) may be involved in activating PKC-ζ. Alternatively, PKC-ζ may have constitutive activity that is resistant to the inhibition of PI3K as indicated in other cell types [36], [37]. This basic level of PKC-ζ activation may be required for HB9 expression in FL-primed hNSCs, whereas the high level of Akt activation in ELL-primed cells negatively regulates the activity of PKC-ζ and thus reduces HB9. On the other hand, we found that PKC-ζ also inhibited Akt activation. Thus, the crosstalk between Akt and PKC-ζ through mutual inhibition of each other may fine-tune the levels of PI3K/Akt/GSK3β activation, which influence the fate of MN differentiation (Fig. 7H).

Defining a negative role of the PI3K/Akt/GSK3β in spinal MN specification from hNSCs allowed us to obtain nearly a pure population of HB9^+^ cells in vitro by inhibiting PI3K activity. The generation of a large quantity of HB9 MNs from hNSCs within only two weeks, much shorter than needed for derivations from embryonic stem cells [38], is important for creating a reliable and efficient in vitro supply of human MNs for pathological studies and drug screen. More importantly, combination of FGF priming together with PI3K inhibition significantly increased the percentage of hNSCs becoming HB9^+^ and ChAT^+^ MNs after transplantation into adult rat spinal cord. Previously, we found that FGF-primed hNSCs became MNs in a region-specific manner, i.e., only in the ventral horn of spinal cord [3], [39]. In this study, however, we observed large HB9^+^/ChAT^+^ MNs from grafted hNSCs in the Lamina V, VI and VIII regions that are reported to lack endogenous ChAT^+^ neurons larger than 25–36 µm [40], [41], indicating that manipulation of the PI3K signaling pathway enables grafted hNSCs to ignore the host environmental cues/limitations. This raises hope that we may be able to find a way to induce endogenous neural stem cells to become MNs in injured or degenerated spinal cords that normally inhibit MN differentiation. Following injury, most tissues tend to enter a repair process in which the PI3K/Akt pathway is critical for enhancing proliferation and survival of various cell types, including NSCs and neurons [42], [43]. NSCs are known to exist in the ependymal layer of the central canal of adult spinal cords [44], [45], but seem to lose their capability to become MNs after spinal cord injury or degeneration. A possible explanation, as indicated by our observation of the negative effect of PI3K/Akt/GSK3β on MN specifications, is that insults trigger the spinal cord cells to elevate their survival mechanisms through PI3K signaling at the expense of inhibiting specific MN differentiation. Questions remain to be answered as whether and how manipulating the PI3K pathway could induce adult spinal cord NSCs to become MNs, as a way of self repair in patients suffering from spinal cord injury or MN diseases.

## Materials and Methods

A detailed description of the materials and methods is found in the online Supporting Information – Supplemental Methods S1.

### Ethics statement

All animal studies were carried out in strict accordance with the ARRIVE guidelines. The protocol (#9910051) was approved by the Institutional Animal Care and Use Committee at the University Of Texas Medical Branch Galveston. All surgeries were performed under isoflurane anesthesia with all efforts made to minimize suffering.

### Human neural stem cell culture, priming and differentiation

Human fetal cortical neural stem cells (hNSCs) were propagated as neurospheres in the presence of EGF, FGF2, and LIF as previously described [2]. For priming, cells were allowed to attach to laminin-precoated surface under either FGF2/heparin/laminin (FHL), EGF/LIF/laminin (ELL), or N2/laminin (N2). For *in vitro* differentiation, primed cells were cultured in B27 containing media.

### Inhibitor treatments and cell viability assays

Human NSCs were treated with specific inhibitors at different doses (Table S1) for various times as indicated in the Results. Cell viability was assessed using a commercially available WST-1 kit according to the manufacturer's instruction (Roche Applied Science, Indianapolis, IN, USA).

### Short hairpin RNA knockdown and induced transgene overexpression

Sigma Mission® shRNA plasmids were screened and used to knockdown the expression of human p110α, p110β or GSK3β genes in hNSCs through Nucleofection or Lentiviral (LV) transduction as described in Supplemental Experimental Procedures. Inducible LVcaGSK3β was constructed based on pLK0.1puro (Sigma), HA GSK3β S9A pcDNA3 (AddGene Inc., Cambridge, MA, USA) and RheoSwitch Mammalian Inducible Expression System (New England Biolabs, Ipswich, MA, USA). Constitutively active GSK3β was over-expressed in hNSCs following LV vector transduction and RheoSwitch Ligand 1 (RSL-1) treatment as described in Supplemental Methods S1.

### Animals and spinal cord transplantation

The ARRIVE guidelines were followed for the animal experiments. FHL-primed hNSCs, with or without PI3K inhibitor LV294002 treatment, were stereotaxically grafted into the L3-4 spinal cord of adult male Sprague Dawley rats according to our previous description [3], [39], [46] and Supplemental Methods S1.

### RNA analyses

Total RNA was extracted and subjected to semiquantitative RT-PCR as previously described [9]. Primer sequences for human GAPDH, HB9, Olig2, Islet1, p110α, p110β and GSK3β are listed in Table S3.

### Western blot analyses

Proteins were extracted at various time points after priming, and subjected to Western blot analyses as described in Supplemental Experimental Procedures. Specific primary antibodies are listed in Table S2.

### Immunostaining and imaging

Fixed cells or tissue sections underwent immunofluorescent staining using antibodies against HB9, Islet1, Olig2, Ngn2, MAP2 or ChAT as described in Supplemental Experimental Procedures and Table S2. Images were acquired with either Nikon 80i epifluorescent microscope or Nikon D-Eclise C1 confocal system.

### Statistical analyses

All analyses included at least three independent experiments unless otherwise stated. For immunofluorescent staining on cultured cells, specific labeling was counted from 10 randomly selected fields of three or four coverslips for at least 1000 total cells per treatment. For *in vivo* studies, at least 200 GFP-labeled hNSCs in each group of five rats per group were counted and percentages of GFP/ChAT double-labeled cells were calculated. All statistical analyses were performed using Student's *t* test, one-way or two-way ANOVA with the aid of GraphPad Prism software (GraphPad Software, Inc. CA, USA).

## Supporting Information

Figure S1 **Effect of manipulating the PI3K/Akt/GSK3β pathway on HB9 levels and cell viability.** (A) Effect of PI3K knockdown on HB9 expression in hNSCs assessed by semi-quantitative RT-PCRs. Cells were transfected with either an empty pLK0.1 vector (control) or with pLK0.1 containing DNA coding for a shRNA targeting the p110β catalytic subunit of PI3K (shP110β). shP110β decreased p110β but had no effect on HB9 transcripts. GAPDH is shown as an internal control. (B) Cell viability in FHL-primed hNSCs assessed by WST-1 four days after treatment with various inhibitors (n = 6). Control, DMSO; WM, wortmannin; LY, LY294002; AKTi5, Akt inhibitor V; AKTi8, Akt inhibitor VIII; Lith, lithium acetate; GSK3i8, GSK3β inhibitor VIII; PS, myristoylated PKC-ζ pseudosubstrate inhibitor. (C) Effect of PI3K inhibition on HB9 mRNA expression in hNSCs assessed by semi-quantitative RT-PCRs. Cells were primed for four days in FHL and treated with the PI3K inhibitor wortmannin (WM, 1 µM). Wortmannin increased HB9 transcripts as compared with the DMSO control. HB9 values were normalized to GAPDH internal controls. (D) Effect of wortmannin (WM) on phosphorylation of Akt in hNSCs assessed by western blot analyses. Cells were primed for 1 day in FHL and treated with the PI3K inhibitor wortmannin (WM, 1 µM). Densitometric values are shown as a ratio of phosphorylated Akt Ser473 (pAkt) over total Akt protein (n = 3). (E) Effect of Akt inhibition on HB9 mRNA expression in hNSCs assessed by semiquantitative RT-PCR analyses. Cells were primed for four days with FHL and treated with various doses of Akt inhibitor V (AKTinh V) (n = 3). (F) and (G) Differential effects of LY294002 at different doses on the phosphorylation of Akt (pAkt) in hNSCs primed with FL and ELL, respectively (n = 3). (H) The levels of total Akt remain unchanged in hNSCs under different treatments (n≥3). All data are presented as mean ± SEM, * p<0.05, ** p<0.01, *** p<0.001, Student's *t* test or ANOVA with post tests.
(EPS)

Figure S2 **Efficient induction of transgene expression by a RheoSwitch inducible system in hNSCs.** A cell line of hNSCs, stably transfected with the pNEBR-R1 plasmid containing the RheoReceptor-1 and RheoActivator genes, was transfected with the pNEBR-X1GLuc vector and stimulated with the RSL-1 ligand 24 h and 48 h after transfection. Four days after the first RSL-1 exposure, conditioned media from these cells was tested for luciferase activity. Treatment with RSL-1 significantly increased the amount of secreted luciferase as compared to the RSL-1 unstimulated control group. Luciferase activities are expressed in arbitrary units, Random luminometer units, and presented as mean ± SEM; n = 3, *** p<0.001.
(PDF)

Figure S3 **Grafted hNSCs with PI3K inhibition ignore environmental cues and differentiate ectopically into large motor neurons in the dorsal horn of adult rat spinal cords.** (A) A representative low magnification confocal image of eGFP-labeled hNSCs becoming choline acetylcholine (ChAT) immunoreactive cells in the Lamina V–VII one month after transplantation into rat spinal cords. Nuclei are counterstained with DAPI. C, central canal. (B–D) Higher magnification images of the inset in (A). Arrow, grafted eGFP^+^ hNSCs are immunofluorescently stained with ChAT^+^ antibodies. Arrowhead, endogenous ChAT^+^ interneurons. Scale bars = 50 µm.
(EPS)

Table S1 **List of the inhibitors.**
(PDF)

Table S2 **List of antibodies.**
(PDF)

Table S3 **List of primers.**
(PDF)

Methods S1 Human neural stem cell expansion, priming and differentiation. Short hairpin RNAs and Nucleofection. Inducible Lentiviral vector construction, packaging and transduction. Animals and transplantation. RNA analysis. Western blot analyses. Immunostaining and imaging.
(DOC)
