# Supplementary figures and images for: Critical Role of PI3K/Akt/GSK3β in Motoneuron Specification from Human Neural Stem Cells in Response to FGF2 and EGF
Source: PLoS One. 2011 Aug 24;6(8):e23414. doi: 10.1371/journal.pone.0023414 (PMC3160859; doi:10.1371/journal.pone.0023414)

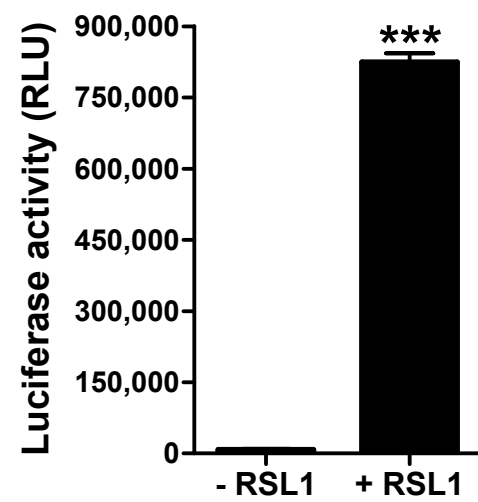

Supplement: Figure S2 — Efficient induction of transgene expression by a RheoSwitch inducible system in hNSCs. A cell line of hNSCs, stably transfected with the pNEBR-R1 plasmid containing the RheoReceptor-1 and RheoActivator genes, was transfected with the pNEBR-X1GLuc vector and stimulated with the RSL-1 ligand 24 h and 48 h after transfection. Four days after the first RSL-1 exposure, conditioned media from these cells was tested for luciferase activity. Treatment with RSL-1 significantly increased the amount of secreted luciferase as compared to the RSL-1 unstimulated control group. Luciferase activities are expressed in arbitrary units, Random luminometer units, and presented as mean ± SEM; n = 3, *** p<0.001. (PDF) [file pone.0023414.s002.pdf]
