# Supplementary material for: Critical Role of PI3K/Akt/GSK3β in Motoneuron Specification from Human Neural Stem Cells in Response to FGF2 and EGF
Source: PLoS One. 2011 Aug 24;6(8):e23414. doi: 10.1371/journal.pone.0023414 (PMC3160859; doi:10.1371/journal.pone.0023414)
Supplement: Table S1 — List of the inhibitors. (PDF) [file pone.0023414.s004.pdf]

Table S1. List of the inhibitors.

INHIBITOR	SOURCE	CONCENTRATION
LY 294002 (PI3K inhibitor)	EMD Chemicals/Calbiochem	0.2, 1, 5, 25 μM
Wortmannin (PI3K inhibitor)	Sigma	0.08, 0.1, 0.2, 0.5, 1 μM
Akt inhibitor V	EMD Chemicals/Calbiochem	0.1, 1, 10 μM
Akt inhibitor VIII	EMD Chemicals/Calbiochem	0.5, 2, 8 μM
Lithium acetate (GSK3β inhibitor)	Sigma	1, 2, 3, 4, 8 mM
GSK3β inhibitor VIII	EMD Chemicals/Calbiochem	1, 2, 10, 20 μM
U0126 (MEK1/2 inhibitor)	Cell Signaling Technology	2 μM
PKCζ pseudosubstrate inhibitor, myristoylated	EMD Chemicals/Calbiochem	1.25, 2.5, 3, 5, 10 μM
GF 109203X (inhibitor for classical and novel PKCs)	Sigma	0.014, 0.5, 1, 2 μM
U73122 (PLCγ inhibitor)	Sigma	1, 3, 10, 15 μM
