# Supplementary material for: Critical Role of PI3K/Akt/GSK3β in Motoneuron Specification from Human Neural Stem Cells in Response to FGF2 and EGF
Source: PLoS One. 2011 Aug 24;6(8):e23414. doi: 10.1371/journal.pone.0023414 (PMC3160859; doi:10.1371/journal.pone.0023414)
Supplement: Table S2 — List of antibodies. (PDF) [file pone.0023414.s005.pdf]

Table S2. List of antibodies.

Name	Type	Vendor	Dilution or Final concentration
Akt	Rabbit polyclonal	Cell Signaling Technology	1:1000
Akt phospho-Ser473	Rabbit polyclonal	Cell Signaling Technology	1:1000
ChAT	Goat polyclonal	Chemicon	1:100
GSK-3β	Rabbit polyclonal	Santa Cruz Biotechnology	1:300
GSK3β phospho-Ser 9	Rabbit polyclonal	Santa Cruz Biotechnology	1:300
GSK3β phospho-Tyr216	Rabbit polyclonal	Santa Cruz Biotechnology	1:200
HB9	Mouse monoclonal	Developmental Studies Hybridoma Bank	1:100
MAP2	Rabbit polyclonal	Chemicon	1:1000
p44/42 MAP kinase	Rabbit polyclonal	Cell Signaling Technology	1:1000
p44/42 MAP kinase phospho Thr202/Tyr204	Rabbit polyclonal	Cell Signaling Technology	1:1000
PKC-ζ	Rabbit polyclonal	Santa Cruz Biotechnology	1:300
PKC-ζ phospho-Thr410	Rabbit polyclonal	Santa Cruz Biotechnology	1:300
RSK Pan	Rabbit polyclonal	R&D Systems	0.5 µg/mL
RSK phospho-Ser380	Rabbit polyclonal	R&D Systems	0.1 µg/mL
