# Supplementary material for: Critical Role of PI3K/Akt/GSK3β in Motoneuron Specification from Human Neural Stem Cells in Response to FGF2 and EGF
Source: PLoS One. 2011 Aug 24;6(8):e23414. doi: 10.1371/journal.pone.0023414 (PMC3160859; doi:10.1371/journal.pone.0023414)
Supplement: Table S3 — List of primers. (PDF) [file pone.0023414.s006.pdf]

Table S3. List of primers.

Name of gene	Forward primer (5' to 3')	Reverse primer (5' to 3')	Annealing temperature (ºC)	Size of product (bp)
GAPDH	TGAAGGTCGGaGTCAACGGA	GATGGCATGGACTGTGGTCAT	60	533
GSK3β	GAGAACTGGTCGCCATCAAGAAAG	ATTGGGTTCTCCTCGGACCAG	60	404
HB9	AGCTGGGCGCCGGCACCTTCC	CCGCCGCCGCCCTTCTGTTTCTC	65	349
Islet1	GCAGCATCGGCTTCAGCAAG	GTAGCAGGTCCGCAAGGTG	58	356
Olig2	GGAGCGAGCTCCTCAAATC	GCTCTGTCATTTGCTTCTTGTC	55	400
p110α	AAGGGCAAATAATAGTGGTG	TTGGCAGTTGAGAATAAAGG	54	336
p110β	TCCTCTTCCATTACCACCAA	ATCGTATTTACCCACGCTAC	54	399
