# Supplementary material for: Critical Role of PI3K/Akt/GSK3β in Motoneuron Specification from Human Neural Stem Cells in Response to FGF2 and EGF
Source: PLoS One. 2011 Aug 24;6(8):e23414. doi: 10.1371/journal.pone.0023414 (PMC3160859; doi:10.1371/journal.pone.0023414)
Supplement: Methods S1 — Human neural stem cell expansion, priming and differentiation. Short hairpin RNAs and Nucleofection. Inducible Lentiviral vector construction, packaging and transduction. Animals and transplantation. RNA analysis. Western blot analyses. Immunostaining and imaging. (DOC) [file pone.0023414.s007.doc]

**Methods S1.**

**Human neural stem cell expansion, priming and differentiation**

Human cortical neural stem cells (hNSCs), lines K048, K054 or G010, were initially derived from 9-11 weeks of human fetuses and provided by Dr. C.N. Svendsen [1]. These cells were expanded as neurospheres in basic medium supplemented with N2, 20 ng/ml recombinant human EGF (R&D Systems, Minneapolis, MN, USA), 20 ng/ml bFGF (R&D Systems), and 10 ng/ml LIF (Chemicon, Temecula, CA, USA) and 5 µg/ml of heparin (Sigma), as previously described [2]. The basic medium consists of DMEM (high glucose, L-glutamine)/Hams-F12 (3:1) (Invitrogen/GIBCO, Carlsbad, CA), 15 mM HEPES (Sigma, St. Louis, MO, USA), 1.5% D-glucose (Sigma), 67 I.U./ml/67 μg/ml penicillin/streptomycin (Cellgro, Herndon, VA) and 2 mM L-glutamine (Sigma). N2 contains 25 µg/ml bovine insulin (Sigma), 100 µg/ml human transferrin (Sigma), 100 µM putrescine (Sigma), 20 nM progesterone (Sigma) and 30 nM sodium selenite (Sigma) [3].

For growing purposes, cells were passaged every 9-10 days by enzymatic and mechanical dissociation into single cells. For priming, hNSCs were allowed to form small spheres for 3-4 days after passage, then either 2 × 10^6^ or 2 × 10^5^ hNSCs were seeded in a T25 flask or 12 mm Carolina Science & Math cover glass circle (Fisher Scientific, Pittsburgh, PA, USA), respectively. All surfaces were precoated with 0.01% poly-D-lysine (Sigma) and 1 μg/cm^2^ mouse laminin (Invitrogen/Gibco, Carlsbad, CA, USA). Cells were incubated with either N2 priming media containing 1 μg/ml laminin; FHL priming media containing 10 ng/ml bFGF, 2.5 μg/ml heparin (Sigma), and 1 μg/ml laminin; or ELL media containing 20 ng/ml EGF, 10 ng/ml LIF, and 1 μg/ml laminin. Cells in T25 flasks were primed for various times as indicated in the results section and collected for detections of specific mRNAs or proteins. Cells on glass coverslips were allowed to further differentiate in B27 medium for 9-10 days before fixation for immunostaining.

**Short hairpin RNAs and Nucleofection**

Mission® shRNA bacterial glycerol stocks targeting human p110α (or PIK3CA, NM_006218), p110β (or PIK3CB, NM_006219) and GSK3β were purchased from Sigma. Plasmid DNAs were amplified and isolated using Plasmid DNA Purification Kits (QIAGEN, Valencia, CA, USA). To identify the shRNAs with the best knock down and specificity for their target, hNSCs were transfected with Endotoxin-Free shRNA containing plasmids using the NSC Nucleofector® Kit according to the manufacturer’s instruction (Lonza/amaxa Inc., Walkersville, MD, USA). Briefly, 5×10^6^ dissociated hNSCs, mixed with 100 µl Nucleofector solution and 6 µg plasmid DNA, were electroporated in the Nucleofector^TM^ Device (Amaxa), and then cultured in humidified 8.5% CO_2_ incubator at 37°C. The transfection efficiency for hNSCs was typically around 40%. To eliminate the cells not expressing the shRNA-plasmid, transfected cells were treated with 0.2-0.4 μg/ml puromycin (Cellgro/Mediatech, Inc., Manassas, VA, USA) 48-72 hrs after transfection. Cells were then cultured for 5-7 more days before RNA collection and RT-PCR. The plasmids, showing over 50% of knockdown efficiency in the initial screening trial, were used in later experiments. The validated specific oligonucleotide sequences are:

p110 α (or PIK3CA, TRCN0000039605)

CCGGCGAGACATTGACAAGATTTATCTCGAGATAAATCTTGTCAATGTCTCGTTTTTG

p110β (or PI3KCB, TRCN0000010025)

CCGGCGACAAGACTGCCGAGAGATTCTCGAGAATCTCTCGGCAGTCTTGTCGTTTTT

GSK3β (TRCN0000000822): CCGGCCCAAATGTCAAACTACCAAACTCGAGTTTGGTAGTTTGACATTTGGGTTTTT

**Inducible Lentiviral vector construction, packaging and transduction**

The inducible Lentiviral vector (LV), LVcaGSK3β, was established based on the RheoSwitch Mammalian Inducible Expression System (New England Biolabs). The RheoSwitch inducible system is composed of two plasmids: pNEBR-R1 and pNEBR-X1. pNEBR-R1 encodes two proteins, RheoReceptor-1 (an engineered ligand-binding domain of an EcR nuclear receptor fused to the yeast GAL4 DNA binding domain) and RheoActivator (an insect/mammalian RXR hybrid ligand-binding domain fused to the viral activation domain VP16), which dimerize to make a holoreceptor. A stable line of hNSCs expressing pNEBR-R1 was first established and validated for its response to the synthetic RheoSwitch Ligand 1 (RSL-1) – induced luciferase expression after pNEBR-X1GLuc transfection.

To construct a LV-based inducible plasmid, pNEBR-X1 of the RheoSwitch system was first engineered to produce pLKX. Briefly, the pNEBR-X1 fragment containing a SV40 terminator, five tandem repeats of the GAL4 response element (5X RE), TATA box, multiple cloning site and SV40 poly A, was PCR-amplified with ClaI and AgeI linkers. The amplified fragment was then cloned into a LV plasmid, pLK0.1puro (Sigma), resulting in pLKX. The constitutively active GSK3β (caGSK3β) cDNA from plasmid 14754: HA GSK3 beta S9A pcDNA3 (AddGene Inc.) was PCR-amplified with SalI linkers and cloned into SalI-linerized pLKX. The resulted pLKXcaGSK3β was then prepared to make LVcaGSK3β.

LV packaging was performed based on the method described previously [4] with modifications. Briefly, human embryonic kidney 293 cells were cultured in 150 mm dishes with DMEM/10% fetal bovine serum at 37˚C, 5% CO_2_. As soon as reaching 80% confluence, cells were co-transfected with a mixture of packaging plasmids (pMDLg/pRRE, CMV-VSVG and RSV-Rev, provided by Dr. Jingwu Xie, UTMB) and the plasmid of interest using the calcium phosphate precipitation method. Two days after transfection, all media were collected, centrifuged at1,500 x *g* for 5 min and filtered through Nalgene low protein binding filters (Fisher Scientific). After centrifugation at 106,750 x g (Optima^TM^ L-80 XP ultracentrifuge, Beckman) and 4˚C for 90 minutes, each pellet was mixed with 100 µl of cold Hanks solution and incubated at 4˚C for at least 12 hours for complete dissolution. The resulting viral stocks were titrated by Quicktiter ^TM^Lentivirus Quantitation Kit (Cell Biolabs Inc., San Diego, CA, USA) according to the manufacturer’s instruction.

# For transduction, dissociated hNSCs were mixed with LV stocks at a multiplicity of infection (MOI) of 0.5-1 (about 10^3-4^ viral particles per cell) and 2-4 μg/ml ploybrene (Sigma). For the inducible caGSK3β part of the study, transduced hNSCs were allowed to form spheres in the proliferation medium containing EGF, FGF2 and LIF for 3 days. Cells were then primed with FHL or ELL for 4 days with or without 0.5 μM RSL-1 (New England Biolabs) before RNA extraction.

**Animals and transplantation**

Adult 300 gram male Sprague Dawley rats (Harlan Laboratories, Houston, TX, USA) were used for transplantation. FHL-primed hNSCs (as described in the priming procedure above) with or without 1 μM LY294002 were transduced by a recombinant adeno-associated viral vector containing the enhanced green fluorescent protein gene, AAVegfp, and further differentiated in B27 medium for 2 days according to our previous description [5–7]. Dissociated cells with higher than 85% viability were resuspended in basic medium supplemented with B27, 250 U/ml DNase, and 3 nM FK506 (Alexis Corp., Switzerland) at a density of 0.5×10^5^ cells/μl, and then stored on ice until grafting. Rats under isoflurane anesthesia received 1 x 10^5^ hNSCs via stereotactic injection into the ventral horn of L3-4 spinal cords. To minimize possible immune rejection, all animals were treated with immunosuppressor NEORAL cyclosporine (Novartis Pharmaceuticals Corp., East Hanover, New Jersey) at 100 μg/ml in drinking water beginning 2 days prior to grafting and then throughout the course of the experiment.

**RNA analysis**

Total RNA was extracted using the RNAqueous-4PCR kit (Applied Biosystems/Ambion, Austin, TX, USA), followed by cDNA generation using a reverse transcription (RT) kit (Applied Biosystems). Semiquantitative RT-PCR was performed using primers listed in Table S2In all cases, 20 ng of cDNA was used in each PCR reaction, and the resulting gel images were captured by Chemi-Imager 4400 v5.5 and analyzed by densitometry with the AlphaEaseFC (FluorChem 8900) software (Cell Biosciences, Inc./Alpha Innotech, Santa Clara, CA, USA).

**Western blot analyses**

Western blot analysis was performed as described previously [2,8] with minor modifications. Cells in T25 flasks were lysed using Cell Lysis Buffer (Cell Signaling Technology, Danvers, MA, USA) and phenylmethylsulfonyl fluoride (PMSF, Sigma). Protein extracts were quantified using the Bradford method. Denatured protein samples (7.5-15 μg) were electrophoresed in NuPAGE® 4-12% Bis-Tris Gels (Invitrogen), and electrophoretically transferred onto Hybond ECL nitrocellulose membrane (Amersham Biosciences). Specific primary antibodies are listed in Table S3. Horseradish peroxidase-conjugated secondary antibodies were used at the indicated dilutions (GE Healthcare/Amersham Biosciences, Piscataway, NJ, USA). All blots were first probed for phosphorylated proteins, followed by stripping in Restore^TM^ Plus Western Blot Stripping Buffer (Thermo Scientific/Pierce Biotechnology, Rockford, IL, USA) and reprobing for the corresponding unphosphorylated proteins and then for
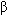
-actin as a loading control. Signals were visualized by ECL^TM^ Plus Western Blotting Detection Reagents (GE Healthcare/Amersham), and analyzed by densitometry with the AlphaEaseFC (FluorChem 8900) software.

**Immunostaining and imaging**

Immunofluorescent staining was performed as previously described [2,5,9]. For *in vitro* study, cells were fixed for 20 min with ice-cold 4% paraformaldehyde, followed by postfixation for 10 min in 100% methanol at –20°C. For *in vivo* study, rats received intracardiac perfusion with 4% paraformaldehyde at 1 month post grafting. Spinal cords were serially cryo-sectioned at 30 μm. Following nonspecific blockage with 5% normal serum+0.3% bovine serum albumin and permeablization with 0.25% Triton X-100, cells were incubated with primary antibodies against ChAT, HB9, or MAP2 at 4°C overnight (Table S3), and then with Alexa fluorophore-conjugated secondary antibodies (1:400, Invitrogen/Molecular Probes, Carlsbad, CA, USA) for 2 h at room temperature in the dark. Cells were counterstained with 1 μg/mL DAPI (Sigma) and then mounted with Fluoromount G (Fisher Scientific). Omission of primary antibodies was used as a control for all markers. All antibodies were empirically tested for optimal concentrations, as well as centrifuged at 13,500×g at 4°C for 2 min to remove any degraded antibodies before use. Images were acquired with a Nikon 80i epifluorescent microscope and the NIS-Elements imaging software (for *in vitro* cells) and with a Nikon D-Eclipse C1 confocal system (for *in vivo* tissue sections).

References

1. Svendsen CN, ter Borg MG, Armstrong RJ, Rosser AE, Chandran S, et al. (1998) A new method for the rapid and long term growth of human neural precursor cells. J Neurosci Methods 85: 141-152.

2. Tarasenko YI, Yu YJ, Jordan PM, Bottenstein J, Wu P (2004) Effect of growth factors on proliferation and phenotypic differentiation of human fetal neural stem cells. J Neurosci Res 78: 625-636.

3. Bottenstein JE, Sato GH (1979) Growth of a rat neuroblastoma cell line in serum-free supplemented medium. Proc Natl Acad Sci U S A 76: 514-517.

4. Dull T, Zufferey R, Kelly M, Mandel RJ, Nguyen M, et al. (1998) A third-generation lentivirus vector with a conditional packaging system. J Virol 72: 8463-8471.

5. Wu P, Tarasenko YI, Gu YP, Huang LYM, Coggeshall RE, et al. (2002) Region-specific generation of cholinergic neurons from fetal human neural stem cells grafted in adult rat. Nat Neurosci 5: 1271-1278.

6. Gao J, Coggeshall RE, Tarasenko YI, Wu P (2005) Human neural stem cell-derived cholinergic neurons innervate muscle in motoneuron deficient adult rats. Neuroscience 131: 257-262.

7. Gao J, Coggeshall RE, Chung JM, Wang J, Wu P (2007) Functional motoneurons develop from human neural stem cell transplants in adult rats. Neuroreport 18: 565-569.

8. Jordan PM, Ojeda LD, Thonhoff JR, Gao J, Boehning D, et al. (2009) Generation of spinal motor neurons from human fetal brain-derived neural stem cells: role of basic fibroblast growth factor. J Neurosci Res 87: 318-332.

9. Gao J, Prough DS, McAdoo DJ, Grady JJ, Parsley MO, et al. (2006) Transplantation of primed human fetal neural stem cells improves cognitive function in rats after traumatic brain injury. Exp Neurol 201: 281-292.
